# Impact of subtrochanteric fractures in the geriatric population: better pre-fracture condition but poorer outcome than pertrochanteric fractures: evidence from the Spanish Hip Fracture Registry

**DOI:** 10.1186/s10195-022-00637-8

**Published:** 2022-03-26

**Authors:** Héctor J. Aguado, Pablo Castillón-Bernal, Paula S. Ventura-Wichner, María C. Cervera-Díaz, Javier Abarca-Vegas, Luis García-Flórez, Jordi Salvador-Carreño, Virginia García-Virto, Clarisa Simón-Pérez, Cristina Ojeda-Thies, Pilar Sáez-López, Juan I. González-Montalvo, RNFC Working Group

**Affiliations:** 1 Orthogeriatric Unit, Servicio de Traumatología y Cirugía Ortopédica, Hospital Clínico Universitario, Av. Ramón y Cajal 3, 47003 Valladolid, Spain; 2 Universidad de Valladolid (UVA), Valladolid, Spain; 3 Trauma Department, Hospital Universitari Mútua de Terrassa Plaza Dr, Robert 5, 08221 Terrasa, Barcelona Spain; 4 Universitat Autònoma de Barcelona (UAB), Bellaterra, Barcelona Spain; 5 Fundación de Investigación HM Hospitales & Fundació Institut d’Investigació en Ciències de la Salut Germans Trias i Pujol (IGTP), Barcelona, Spain; 6 Department of Traumatology and Orthopaedic Surgery, Hospital Universitario 12 de Octubre, Madrid, Spain; 7 Instituto de Investigación del Hospital La Paz, IdiPAZ, Madrid, Spain; 8 Department of Geriatrics, Hospital Universitario Fundación Alcorcón, Madrid, Spain; 9 Head Coordinator of the Spanish National Hip Fracture Registry, Madrid, Spain; 10 Geriatric Medicine Department, Hospital Universitario La Paz, Madrid, Spain; 11 Department of Medicine, Universidad Autónoma de Madrid, Madrid, Spain

**Keywords:** Aged, Geriatric, Hip fracture, Pertrochanteric, Subtrochanteric, Intertrochanteric, Extracapsular, Registry, Outcome, Complication, Fragility fracture

## Abstract

**Background:**

Clinical management in orthogeriatric units and outcome indicators are similar for extracapsular fragility hip fractures, without discriminating between subtrochanteric and pertrochanteric fractures.

**Hypothesis:**

Geriatric patients, 75 years or older, with subtrochanteric fractures have worse clinical and functional outcomes than those with pertrochanteric fractures.

**Materials and methods:**

Retrospective observational study of data prospectively collected by the Spanish Hip Fracture Registry including patients 75 years or older, admitted for extracapsular hip fractures from January 2017 to June 2019. Demographic and baseline status, pre-operative, post-operative and 30-day follow-up data were included.

**Results:**

A total of 13,939 patients with extracapsular hip fractures were registered: 12,199 (87.5%) pertrochanteric and 1740 (12.5%) subtrochanteric. At admission, patients with subtrochanteric fractures were younger (86.5 ± 5.8 versus 87.1 ± 5.6 years old), had better pre-fracture mobility (3.7 ± 2.7 versus 3.9 ± 2.8) (1-to-10 scale, 1 being independent) and were more likely to be living at home; those with pertrochanteric fractures had worse cognitive function (Pfeiffer 3.3 ± 3.3 versus 3.8 ± 3.5). The subtrochanteric fracture group showed worse post-fracture mobility (7.3 ± 2.7 versus 6.7 ± 2.7) and greater deterioration of mobility (3.7 ± 3.0 versus 2.9 ± 2.7). Among individuals living at home at baseline, those with subtrochanteric fractures were more likely to remain in an assisted facility at 30-day follow-up. In-hospital mortality during acute admission was higher for the subtrochanteric group (5.6% versus 4.5%) (*p* = 0.028). To our knowledge, this is the first paper highlighting the differences between these two fracture groups in the geriatric population.

**Conclusions:**

Subtrochanteric fractures in the older population are a different and worse entity, with greater morbimortality and functional decline than pertrochanteric fractures. Despite being younger and fitter at admission, older patients with subtrochanteric fractures have a higher risk of remaining non-weight bearing and undergoing re-operation and institutionalization. Orthogeriatric units should be aware of this and manage subtrochanteric fractures accordingly.

Level of evidence: IV.

**Supplementary Information:**

The online version contains supplementary material available at 10.1186/s10195-022-00637-8.

## Introduction

Extracapsular hip (proximal femur) fractures in older patients are clinically managed similarly in orthogeriatric units and evaluated using the same clinical outcomes, without discriminating between subtrochanteric and pertrochanteric fractures. However, surgical treatment of subtrochanteric fractures is more challenging [1–5]. National hip fracture registries currently collect data differentiating intra- and extracapsular fractures, but no further subgroup analysis is performed [2, 3, 5–12]. Evidence supporting differences in clinical history, outcome and complications between intertrochanteric and subtrochanteric fractures is scarce [2, 13, 14].

The subtrochanteric fractures can extend proximally (trochanteric or femoral neck) or distally (diaphyseal) [4, 15, 16]. They account for approximately 5–22% of all extracapsular hip fractures and have a negative effect on quality of life and social function [17].

Our working hypothesis is that patients in the geriatric population presenting with subtrochanteric fractures have worse clinical and functional outcomes than those with pertrochanteric fractures. Knowledge of the potential differences between both types could raise awareness towards subtrochanteric fractures, reduce complications and improve their outcome.

## Materials and methods

We performed a retrospective observational study of data prospectively collected by the Spanish Hip Fracture Registry (Registro Nacional de Fracturas de Cadera, RNFC) [10, 18] including all patients diagnosed with extracapsular hip fractures from January 2017 to June 2019. The RNFC is a multi-centre, observational, prospective audit including all patients 75 years or older admitted for hip fractures in any of the 77 participating centres, followed up for 30 days, who provide consent [18], registering the variables proposed in the Fragility Fracture Network Minimum Common Dataset (FFN MCD). All participating centres received approval from their local ethics committees, and this study has therefore been performed in accordance with the ethical standards laid down in the 1964 Declaration of Helsinki.

Of all patients treated in the participating centres, 96.5% consented inclusion in the registry. Collaborators from the included hospitals participate and include data on a voluntary basis, following approval by local institutional review boards. The RNFC is registered in the Spanish Data Protection Agency [9]. Extracapsular hip fractures were classified into pertrochanteric or subtrochanteric following the definition proposed by Fielding [15, 16]. Atypical or pathological fractures were excluded from this study.

The RNFC collects data grouped into:

*(1) Demographic and baseline data:* Age, sex, fracture type, side, cognitive status, overall health [American Society of Anesthesiologists (ASA) physical status classification system], mobility, place of residence and osteoprotective treatment. Each patient’s pre-fracture mobility was graded from 1 to 10, (1 “completely independent gait”, 10 “no mobility at all or with the help of 2 other people”) (Table 1). Cognitive status was assessed using Pfeiffer’s Short Portable Mental Status Questionnaire [19]. Osteoprotective treatment was defined as either anti-resorptive (i.e. bisphosphonates) or bone-forming medication.

**Table 1:** Consensus table grading mobility status from 1 to 10

1	Independent mobility out and at home, without technical aids
2	Independent mobility out and at home, with one technical aid
3	Independent mobility out and at home, with two technical aids or walker
4	Independent mobility only at home, without technical aids
5	Independent mobility only at home, with one technical aid
6	Independent mobility only at home, with two technical aids or walker
7	Independent mobility only at home, watched by carer or relative
8	Mobility only at home, with little extra help from carer or relative
9	Mobility only at home, with little extra help from carer or relative
10	Mobility with the help of two people or no mobility

*(2) Operative care:* Time from presentation to surgery (days and hours), type of anaesthesia and surgery, and physician co-management during acute hospitalization. Surgical treatment is divided into internal fixation (i.e. nails, plates or screws), joint replacement (hemi- or total hip arthroplasty, cemented or uncemented), or non-operative management.

*(3) Post-operative care:* Mobilization on the first post-operative day, total and post-operative length of hospital stay, discharge destination, osteoprotective treatment at discharge, pressure ulcers and vital status.

*(4) Thirty-day follow-up:* Mobility (from 1 to 10), osteoprotective treatment, place of residence, re-admission to hospital and re-operations within 1 month, difference between baseline and 1-month mobility, and vital status.

Data were analysed using descriptive statistics. Quantitative results are expressed as mean ± standard deviation. Qualitative or dichotomous variables are expressed in percentages. The chi-squared test was used to compare proportions and study the relationships between them. Comparisons between two quantitative variables were made using Student’s *t*-test. To evaluate relationships, odds ratio (OR) and 95% confidence intervals (95% CI) were calculated. The level of significance was set at *p* < 0.05. All statistical analyses were performed using SPSS v.23 software (IBM, Armonk, NY, USA).

## Results

A total of 13,939 patients with extracapsular hip fractures were registered between January 2017 and June 2019: 12,199 (87.5%) had pertrochanteric fractures and 1740 (12.5%) subtrochanteric fractures.

### *Demographics and baseline data:* (Table 2)

**Table 2:** Demographics and pre-fracture status expressed in mean ± standard deviation and percentage

Demographics and pre-FX status	Pertrochanteric	Subtrochanteric	OR (95% CI)/*p*
Age*	87.1 ± 5.57	86.5 ± 5.77	< 0.001
Female versus male	76.7% versus 23.3%	78.4% versus 21.6%	0.906 (0.803–1.024)/0.115
Right versus left side	51% versus 49%	51.5% versus 48.5%	0.938 (0.889–1.087)/0.736
Pfeiffer*	3.77 ± 3.45	3.33 ± 3.31	< 0.001
ASA	2.83 ± 0.63	2.84 ± 0.64	0.462
Pre-FX mobility*	3.90 ± 2.76	3.74 ± 2.74	0.025
Living at home before fracture*	76.2%	79.4%	1.198(1.058–1.355)/0.004
Osteoprotective treatment pre-FX:*	5.8%	7.9%	1.391 (1.150–1.683)/0.001
Anti-resorptive*	5.4%	7.3%	1.367 (1.122–1.665)/0.002
Bone forming	0.6%	0.8%	1.313 (0.740–2.328)/0.350

Odds ratio (OR) and 95% confidence intervals (95% CI). *p < 0.05

Patients with extracapsular hip fractures were predominantly female (76.9%), similarly for pertrochanteric and subtrochanteric fractures. Those admitted for subtrochanteric fractures were significantly younger (86.5 ± 5.8 versus 87.1 ± 5.6 years old), had better pre-fracture mobility (3.7 ± 2.7 versus 3.9 ± 2.8) and were more likely to be living at home than in nursing homes (76.2% versus 79.4%). Those with pertrochanteric fractures had worse cognitive function at admission (Pfeiffer 3.3 ± 3.3 versus 3.8 ± 3.5).

### *Operative care:* (Table 3)

**Table 3:** Operative care data expressed in means ± standard deviation and percentages

Operative care	Pertrochanteric	Subtrochanteric	OR (95%CI) / p
Time (days) to operation	2.78 ± 4.56	2.98 ± 3.37	0.078
Time (hours) to operation	66.28 ± 108.92	69.64 ± 65.99	0.215
Type of anaesthesia			
General	6.4%	6,9%	1.083 (0.884–1.327)/0.442
Neuro-axial	93,6%	93.1%	
Surgical Treatment*			
Fixation	97.5%	96%	< 0.001
Joint replacement	1.8%	3.2%	
Non-operative	0.7%	0.8%	
Clinical assessment			
Geriatrician	76.3%	74.6%	0.637
Internal medicine	19.4%	21.2%	
Both	0.6%	0.5%	
Only trauma surgeon	3.6%	3.7%	

Odds ratio (OR) and 95% confidence intervals (95%CI). *p < 0.05

There were no differences between groups regarding surgical delay (2.8 ± 4.6 versus 3.0 ± 3.4) or type of anaesthesia used (neuro-axial 93.6% versus 93.1% or general 6.4% versus 6.9%) (*p* = 0.442). Intramedullary nailing was the main type of internal fixation for both groups, with a minority treated with plates. Only 96 patients (0.7%) were treated non-operatively. The proportion of fractures managed with arthroplasty was higher for subtrochanteric fractures than for pertrochanteric fractures (3.8% versus 1.8%). Orthopaedic surgeons performed clinical assessment and follow-up during acute admission, and the proportion with involvement of geriatricians (76.3% versus 74.6%) and/or internal medicine specialists (19.4% versus 21.2%) was similar for both groups (*p* = 0.637).

### *Post-operative care and hospital discharge:* (Table 4)

**Table 4:** Post-operative care data expressed as mean ± standard deviation and percentage

Post-operative care	Pertrochanteric	Subtrochanteric	OR (95% CI)/*p*
Out of bed < 24 h post-operation*	64.7%	59.5%	1.252 (1.128–1.389)/< 0.001
Post-operative length of hospital stay (days)*	7.61 ± 5.61	8.49 ± 7.20	< 0.001
Total length of hospital stay (days)*	10.20 ± 6.47	11.32 ± 7.85	< 0.001
Pressure ulcers	6.1%	6.7%	0.349
Hospital discharge:			1.032 (0,928–1.147)/0.563
Own home or family	40.5%	39,7%	
Healthcare facility	59.5%	60.3%	
Bone-protective treatment discharge	40.4%	40.4%	1.001 (0.901–1.112)/0.986
Anti-resorptive	38.7%	38.1%	1.026 (0.922–1.141)/0.640
Bone forming	2.5%	3.1%	0.800 (0.591–1.082)/0.147

Odds ratio (OR) and 95% confidence intervals (95% CI). *p < 0.05

Patients with subtrochanteric fractures were less likely to be mobilized on the first post-operative day (*p* < 0.001). Both total and post-operative length of stay were longer for subtrochanteric fractures (*p* < 0.001). No differences regarding discharge destination could be found between groups (*p* = 0.572).

### Thirty-day follow-up, functional outcome, complications and mortality rates: (Table 5)

**Table 5:** Thirty-day follow-up data expressed as mean ± standard deviation and percentage

Thirty-day follow-up	Pertrochanteric	Subtrochanteric	OR (95% CI)/*p*
Alive at 30-day follow-up	92.1%	91.0%	1.148 (0.960–1.374)/0.130
No weight bearing within 30 days post-operation*	8.9%	22.3%	0.338 (0.256–0.445)/< 0.001
Post-FX mobility (30 days)*	6.66 ± 2.67	7.33 ± 2.71	< 0.001
Pre-FX versus post-FX mobility (30 days)*	2.88 ± 2.69	3.72 ± 2.98	< 0.001
Living at own home after 30 days*	48.3%	44.8%	0.864 (0.775–0.962)/0.008
Living in own home pre-fracture but at healthcare facility after 30 days*	38.0%	44.5%	< 0.001
Bone-protective treatment:	42.5%	42.3%	1.009 (0.905–1.124)/0.887
Anti-resorptive	39.7%	38.7%	1.046 (0.938–1.168)/0.419
Bone forming*	3.6%	4.8%	0.747 (0.578–0.965)/0.025
Hospital re-admission within 30 days post-operation	3.1%	2.5%	1.206 (0.869–1.673)/0.263
Re-operation < 30 days post-operation*	1.7%	1.9%	0.011
Wound surgical revision*	0.5%	1.1%	2.418 (1.413–4.137)/0.001
Hip arthroplasty dislocation	0.2%	0.1%	
Failed fixation	0.7%	0.4%	
Peri-implant fracture	0.1%	0.2%	
Other reasons	0.2%	0.1%	

Odds ratio (OR) and 95% confidence intervals (95% CI). *p < 0.05

Weight bearing was less likely to be permitted in patients with subtrochanteric fractures (*p* < 0.001), who also showed worse post-fracture mobility (*p* < 0.001), and a greater deterioration of baseline mobility versus 30-day mobility (*p* < 0.001). The proportion of patients living in assisted facilities at 1 month was higher among those with subtrochanteric fractures (*p* = 0.008). Among patients living at home before the fracture, those with subtrochanteric fractures had a higher risk of remaining in an assisted facility at 1 month: 44.5% were unable to return home (OR 37.31, 95% CI 19.67–70.77; *p* < 0.001) versus 38.0% for the pertrochanteric group (OR 46.37, 95% CI 37.18–57.83; *p* < 0.001). Patients with subtrochanteric fractures were more likely to receive bone-forming medication (*p* = 0.025). The re-operation rate was higher among those with subtrochanteric fractures, most commonly for revision of the surgical wound. Other indications for re-intervention were: prosthetic hip dislocations, failure of internal fixation, and peri-implant fracture (around a prosthesis or an internal fixation device).

Mortality during acute hospitalization was higher for the subtrochanteric fracture group (*p* = 0.028) (OR 1.27, 95% CI 1.03–1.59). There were no differences in 30-day mortality (subtrochanteric group 91.0% survival versus 92.1% for the pertrochanteric group) (*p* = 0.130). In a subgroup analysis according to the surgical treatment, there were no differences in mortality at any time of follow-up (*p* = 0.545).

## Discussion

We observed large differences between pertrochanteric and subtrochanteric fractures regarding management and outcomes. Patients with subtrochanteric fractures were mobilized later and were more likely to remain non-weight bearing. In spite of a better baseline situation, patients with subtrochanteric fractures had worse 30-day outcomes: they became more dependent, needed more re-operations, had worse mobility and had a higher risk of living in an assisted facility. They also showed greater in-hospital mortality, though this difference was lost at 30-day follow-up.

### Demographic and baseline data

The demographic characteristics of the patients included in the RNFC were similar to those described by other international registries [3, 6, 7, 9, 11, 12]. Patients with subtrochanteric fractures were 6 months younger, although this difference is not clinically relevant. They had better pre-fracture mobility and less cognitive impairment, and were more likely to live at home than patients with pertrochanteric fractures. Patients treated with anti-resorptive medication before the fracture had higher odds of suffering subtrochanteric fractures than pertrochanteric fractures, even though atypical fractures were excluded from this study. We did not observe any differences regarding fracture type among patients treated with bone-forming agents. These findings are consistent with data described in female Medicare beneficiaries: Wang et al. found that use of oral bisphosphonates increased the risk for subtrochanteric and femoral shaft fractures, but not intertrochanteric fractures [20]. Ng et al. observed a slight increase in non-hip femoral fracture rates (including subtrochanteric fractures) in Olmsted County (USA), despite a decreasing hip fracture incidence [21].

### Operative care

Both extracapsular hip fracture types had similar involvement of clinical specialists during acute hospitalization; surgical delay, type of anaesthesia and discharge destination were similar for both groups. However, the type of surgery and the post-operative mobilization protocols differed: more subtrochanteric fractures were treated with hip arthroplasties despite the lack of metaphyseal support for the femoral stem. Reasons for the choice of treatment are not registered by the RNFC. Subtrochanteric fractures are more difficult to fix [4, 8], so many surgeons might choose a hip replacement, which offers more mechanical stability, looking for better mobility, early weight bearing and faster recovery. There were 22 patients with a subtrochanteric fracture treated with a hip arthroplasty: 13 of them were performed at three hospitals, known as referral centres for complex hip replacement revision surgery. In certain scenarios and aiming for early weight bearing, they might feel more comfortable replacing than fixing a subtrochanteric fracture.

Over 96% of patients were assessed and/or followed up by geriatricians and/or internal medicine, with nearly 75% only by geriatricians. Nevertheless, there were no differences in assessment by clinical specialists between both groups: the differences in mortality and functional outcome observed could not be justified by differences in medical care. There was no control group to assess the weight of geriatricians and internal medicine in patient care, as there is no hospital involved in the RNFC with only trauma surgeons managing patients with hip fractures. The type of fracture and the type of surgery seem to determine the worse outcomes observed in subtrochanteric fractures, despite a better baseline status and similar surgical delay and anaesthetic management.

### Post-operative care and hospital discharge

Total and post-operative length of stay in acute hospitalization was 1 day longer for individuals with subtrochanteric fractures. Although the RNFC does not record data that could account for these differences, there are several possible reasons for this delay: blood loss is higher among patients with subtrochanteric fractures. Limitation of weight bearing hinders discharge to home, increasing length of stay. Furthermore, patients with subtrochanteric fractures were mobilized later.

The longer length of stay observed is in line with Karayiannis’s report: patients treated with cerclage cables/wires (mainly used in subtrochanteric fractures) had a longer length of stay [22]. Bandhari et al. identified predictors for a longer length of stay: admission from a long-term care facility, living at home with support, and advanced age, suggesting we should have observed a longer hospital stay among those with pertrochanteric fractures, when quite the contrary was found. Developing complications was the only predictor for length of stay associated with subtrochanteric fractures [23].

Patients with subtrochanteric fractures were mobilized later. This could be due to fear of fracture displacement or failure of fixation, where delaying mobilization gives a false sense of security. Shukla et al. reported that up to 40% of subtrochanteric fractures require open reduction [5]. This involves larger incisions, increasing blood loss and post-operative pain [22]. This could also justify the higher post-operative stay and in-hospital mortality for subtrochanteric fractures [12, 24].

### Thirty-day follow-up, functional outcome and complication

It is remarkable that patients with subtrochanteric fractures had worse 1-month mobility, despite better pre-fracture mobility. Comparison of pre-fracture and 30-day mobility for each patient showed a greater decline in those with subtrochanteric fractures. Both findings suggest that the functional outcome is worse for patients with subtrochanteric fractures. We found that 22.3% of patients with subtrochanteric fractures (versus 8.9% for pertrochanteric fractures) remained non-weight bearing after 30 days, possibly explaining the higher proportion of patients not returning home after 30 days. If patients with a subtrochanteric fracture could bear weight and be discharged to rehabilitation facilities, they would have a greater chance of returning home and having better functional outcomes. Patients with subtrochanteric fractures had more re-operations, particularly revisions of surgical wounds, which can be explained by a higher proportion of open reductions. Surgical complications and non-unions are more common in subtrochanteric fractures than in fractures of other regions of the femur, especially among patients with poor bone quality, unfavourable fracture patterns or suboptimal placement of internal fixation [1, 5, 25–29].

Nearly 60% of the patients in both groups were sent to an assisted facility. Thirty days after hip fracture diagnosis, patients with a subtrochanteric fracture were more likely to remain in an assisted facility. This is a very serious issue: a large number of patients living independently become dependent and are at risk for institutionalization. Our study found some probable reasons: patients remain non-weight bearing longer and have poorer mobility, which hinders living at home. Several solutions could be applied: improvement of surgical techniques including stronger internal fixation and smaller surgical approaches, specialized surgical teams to treat these more difficult fractures, availability of rehabilitation facilities after acute hospitalization and guidelines for the management of these fractures. Failure to research and provide solutions for the functional deterioration following subtrochanteric fractures may lead to a serious public health problem due to increasing institutionalization, which is likely to increase in the near future. All extracapsular hip fractures are grouped together in registries, but subtrochanteric fractures are different from pertrochanteric fractures: both fractures behave differently, with different baseline characteristics, complication rates and outcomes. A more directed management of subtrochanteric fractures might lead to better outcomes.

Furthermore, the data we present are from right before the coronavirus disease 2019 (COVID-19) pandemic, which might impact the presentation and outcome of hip fractures. COVID-19 infection and a concomitant hip fracture presented worse function, with important repercussions for general health [30].

Our study has several strengths. We included a large number of patients, which is representative of the country’s population. As Spain has a population similar to that of many other Mediterranean or European countries, these results can be applied to other regions. This is, to our knowledge, the first study providing evidence on the worse clinical and functional outcomes of the subtrochanteric fractures versus pertrochanteric fractures in the geriatric population.

Our study has also several limitations inherent to national registries. First, not collecting variables in more detail limits analysis; more variables could help with interpreting of the results of this study. Second, there is a confounding factor as not all cases are treated the same way in the different hospitals. Third, the RNFC is mainly supported by geriatricians working in trauma units: hospitals with no specialized teams managing patients with hip fractures are not represented. Therefore, we cannot compare their results and weight the importance of the orthogeriatric units managing subtrochanteric fractures. Different risk factors might be responsible for the worse functional outcome of subtrochanteric fractures, including delayed weight bearing or physical deterioration after fracture. Reasons for the indication of non-weight bearing could be poor reduction or unstable fixation, but we were unable to analyse this in greater detail. Future studies should evaluate the influence of delayed weight bearing on functional results, as well as reasons for delayed weight bearing such as quality of reduction, type of fixation or post-operative fracture stability.

In conclusion, pertrochanteric and subtrochanteric fractures in older people are commonly treated similarly. However, geriatricians and orthopaedic surgeons sense that both groups may have different features and outcomes, and our results provide evidence to support this statement. To our knowledge, this is the first study pointing out the differences between extracapsular fractures in older patients. Individuals suffering subtrochanteric fractures are significantly younger, have less cognitive impairment and are more likely to live at home before the fracture, but have a higher risk of remaining non-weight bearing after surgery, with worse functional outcomes, more re-operations and more institutionalization. In-hospital, but not 1-month, mortality was higher. We can thus conclude that, in patients 75 years old and older, subtrochanteric fractures are different from pertrochanteric fractures with a worse prognosis, including higher morbidity, mortality and functional decline. Though both are extracapsular fractures, they should be addressed differently as subtrochanteric fractures present a higher risk of complications.

## Supplementary Information

**Additional file 1.** List members of the RNFC working group.

## Data Availability

The data that support the findings of this study are available from the Spanish Hip Fracture Registry (Registro Nacional de Fracturas de Cadera, RNFC), but restrictions apply to the availability of these data, which were used under license for the current study and so are not publicly available. Data are, however, available from the authors upon reasonable request and with permission of Registro Nacional de Fracturas de Cadera, RNFC.

## Abbreviations

RNFC: *Registro Nacional de Fracturas de Cadera*, The Spanish Hip Fracture Registry
FFN MCD: Fragility Fracture Network Minimum Common Dataset
ASA: American Society of Anesthesiologists
OR: Odds ratio
CI: Confidence interval
