# Supplementary material for: Impact of subtrochanteric fractures in the geriatric population: better pre-fracture condition but poorer outcome than pertrochanteric fractures: evidence from the Spanish Hip Fracture Registry
Source: J Orthop Traumatol. 2022 Mar 26;23:17. doi: 10.1186/s10195-022-00637-8 (PMC8960105; doi:10.1186/s10195-022-00637-8)
Supplement: Supplementary file 1 — Additional file 1. List members of the RNFC working group. [file 10195_2022_637_MOESM1_ESM.docx]

ANNEX 1

Participants in each Hospital: **Hospital Comarcal de Alto Deba. Guipúzcoa-País Vasco:** Uxue Barrena, Amaia Santxez, Iñigo Etxebarria**. Complejo Asistencial de Ávila. Castilla y León**: Flavia Lorena Hünicken,  Natalia Sánchez Hernández. **Consorci Sanitari de Terrassa, Barcelona**: Leonor Cuadra Llopart, Georgina Cerdà Mas, Pedro Zubeldia Tomás,  Anna Soriano Villabi. **Hospital de la Santa Creu i Santpau**: Jordi Martin Marcuello, Joan Sugrañes Camprubi, Laura García-Fontes Pujol. **Centre Sociosanitari El Carme. Badalona-Cataluña**: José Manuel Cancio Trujillo, José Luis Rodríguez García. **Hospital de Manises. Valencia**: José S. Barreda Puchades, Marta Ribelles Férez. **Hospital Virgen del Puerto. Plasencia-Cáceres. Extremadura**: Raquel Ortés Gómez, Estela Villalba Lancho, Guadalupe Lozano Pino, Jean Carlo Heredia Pons. **Hospital Clínico Universitario de Santiago. Galicia:** Eduardo Del Río Pombo, Aurora Freire Romero, María Otero Fernández, Noelia Sende Munín, Carmen Ruanova Quintas. **Hospital d’Olot i Comarcal de la Garrotxa. Girona-Cataluña:** Gemma Badosa,  José Ernesto Matamoros y Hugo Briceño **Hospital Provincial Sagrado Corazón de Jesús. Huesca-Aragón**: Isabel Peralta Gascón, Caterina Soler Frias, Elena Ubis Diez. **Complejo Asistencial Universitario de León. Castilla y León:** Sonia Jiménez Mola, F. Javier Idoate Gil. **Hospital General Universitario Gregorio Marañón. Madrid:** Virginia Mendoza Moreno, Nuria Montero. **Hospital Universitario Fundación Jiménez Díaz. Madrid:** Ana Isabel Hórmigo Sánchez, María Almudena Milán Vegas,  Myriam Rodríguez Couso, Marta García Salmones Fragoso, Virginia Ruiz Almarza, Esther Lueje Alonso, Javier Sánchez Martin, Cristina Trenado Salan. **Hospital Clínico San Carlos. Madrid**: Jesús Mora Fernández, Mijail Méndez Hinojosa, Diana K. Villacrés Estrada, Ana Mª Moreno Morillo, Eduardo Solís Vaqueiro. **Hospital General de Villalba. Collado Villalba-Madrid**: Verónica Martín López. **Hospital Universitario Infanta Sofía. San Sebastián de los Reyes-Madrid:** Marta Neira Álvarez, Ana Hurtado Ortega, Rubén Herreros Ruiz Valdepeñas, Guillermo Carretero Cristobal, Carlos Fagundez García. **Hospital Universitario de Getafe. Madrid:** María Auxiliadora Julia Illán Moyano , Alba Costa Grille. **Hospital Universitario Severo Ochoa. Leganés-Madrid:** María Jesús Molina Hernández. **Hospital Universitario de Móstoles. Madrid:** María del Pilar Rodríguez Sanjuan Agustín Prieto Sánchez, Sonia Nieto Colino, Francisco Javier Cid Abasolo, Inmaculada Boyano Sánchez. **Hospital Universitario de Cabueñes. Gijón-Asturias:** María Luisa Taboada Martínez. **Hospital Vital Álvarez-Buylla. Mieres-Asturias**: Marta Alonso Álvarez, David Bonilla Díez, Abel Martínez Gago, Aroa Roces Fernández. **Hospital Universitario Marqués de Valdecilla. Santander-Cantabria**: Mª Jesús Sanz-Aranguez Ávila, Jesús Pérez del Molino Martín,  Marta Madariaga Canoura,  Zoilo Yusta Escudero. **Complejo Asistencial de Segovia-Castilla y León:** María Teresa Guerrero Díaz, Mari Cruz Macias Montero, Pilar Jesús Del Pozo Tarrago, Elena Ridruejo Gutiérrez de la Cámara, Angélica Muñoz Pascual, Ana Suero y Alberto María Núñez. **Hospital Clínico Universitario de Valladolid-Castilla y León**: M Carmen Cervera Díaz. Virginia García Virto, Héctor J. Aguado Hernández, Ana Zabalza Peláez, Elvira Mateos Álvarez, Adela Pereda Manso. **Hospital Nuestra Señora de Gracia. Zaragoza-Aragón**: Jorge Corrales Cardenal, Claudia Patricia Murillo Erazo, Elisa Lasala Hernández  Nora Molina Torres, Ana Cristina Sanjoaquin Romero. **Hospital General Obispo Polanco. Teruel-Aragón**: Ángel Castro Sauras, Alejanndro Urgel Granados, María Pilar Muniesa Herrero, Vicente Sánchez Ramos, Miguel Ranera García, José Adolfo Blanco Llorca, María Teresa Espallargas Doñate, Silvida Aldabas Soriano, María Royo Agustín, Agustín Rillo Lázaro, Jorge García Fuentes, Alberto Planas Gil. **Hospital Sociosanitario Francolí. Tarragona-Cataluña**: Eugenia Sopena Bert. **Consorci Sanitari Garraf. Barcelona-Cataluña**: Laura  Alexandra  Ivanov, Dealbert  Andrés  Alfred, Macho  Pérez Óscar, Josefa  López  De  La  Fuente.Hospital Reina Sofía. Tudela-Navarra: Pablo Díaz de Rada Lorente, María Sanz Gávez, Ignacio Gómez Ochoa, Lucas Eza Moreno, Daniel Sánchez Zalabardo, Noemi Borobia Rodríguez, Julián Díaz Jiménez, Aitziber Echeverría Echeverría, María Rosa González Panisello, Isabel Irigoyen Urroz, Eva Marta Miñana Barrios, Rosa Orta Álava, Sara Pinilla Baigorri, Jon Ros Añón, Lucía Zardoya Arcega, Maite Fuste Angos. **Hospital Valle de los Pedroches. Pozoblanco. Córdoba-Andalucía:** Manuel Mesa Ramos, Pilar Márquez de Torres, María del Mar Higuera Álvarez de los Corrales. **Hospital El Pilar. Barcelona-Cataluña:** Silvia Comas. **Hospital Universitario Cruces. Barakaldo-País Vasco:** Rocio Prieto Martín, Borja Villarejo Fernández, Esteban Javier Aragon Achig, Nerea Hernández González, Iraia Arteagoitia Colino, Josu Merino Pérez. **Hospital Universitario Puerta de Hierro. Majadahonda-Madrid:** Cristina Bermejo Boixareu, Jesús Campo Loarte,  Gema Piña Delgado,  Macarena Díaz de Bustamante De Ussía,  Armando Pardo Gómez,  Iluminada Martín García,  Ainhoa Guijarro Valtueña, Fernando Segismundo Jañez Moral, Samuel González González, Juan Martínez Candial. **Consorci Sanitari de Terrassa – Hospital de Terrassa. Barcelona-Cataluña:** Laura Puertas, Pablo Castillón, Cristina Estrada, Olga Gómez, Irene Omiste, Laura Rey, Carlos García. **Hospital Regional Universitario de Málaga. Andalucía**: Verónica Pérez Del Río. **Hospital del Henares. Coslada-Madrid:** Sonia Bartolomé, Francisco Coca. **HLA Clínica Vistahermosa. Alicante-Comunidad Valenciana:** Javier Sanz Reig, Jesús Más Martínez**.** **Complejo Hospitalario Universitario de Albacete. Castilla-La Mancha:** Amalia Navarro Martínez, Francisco Medrano González, Lourdes Sáez Méndez, Virginia Parra Ramos, Crsitina Rosa Felipe, Mº Cortes Avilés Martínez, Sergio Losa Palacios, Joaquín Alfaro Micó, Ainara Achaerandio de Nova, Mª del Carmen Viejobueno Mayordomo, Leticia García Sánchez, Noelia Ramayo Díaz. **Hospital La Luz – Grupo Quirónsalud. Madrid**: Ignacio Maestre. **Hospital Vega Baja Orihuela – Alicante. Comunidad Valenciana**: José Eduardo Salinas Gilabert, Vicente Mira Viudes, Javier Rincón Recarey, Joaquín Ortuño Moreno, Antonio Ortín Barcelo, Miguel Ángel Palazón Banegas, Beatriz Muela Pérez, Francisco Navarro Gonzalvez, Silvia Correoso Castellanos, Alberto García Gálvez, Ana Corraliza Zamorano, David Coves Mojica, Elena Blay Domínguez, Eva María Veracruz Gálvez, María Luz Aguilar Martínez, Pilar Benito Muñoz, María del Carmen Rosa Delgado, María del Carmen González Jara, Jesús Jiménez Olivares, David Hernández Lozano. **Hospital Universitario de Basurto. Vizcaya-País Vasco:**Unai García De Cortázar Antolín, Mirentxu Arrieta Salinas, Daniel Escobar Sánchez, Estibaliz Castrillo Carrera, Josu Lauzirika Uranga, Mar Abeal López, Javier Hoyos Cillero, Ainara Izaguirre Zurinaga, Cesar García Puertas, Arkaitz Lara Quintana, Borja Cuevas Martínez, Andrea Dominguez Ibarrola, Julia Isabel Martino Quintela, Idoia Villamor García, Ander Moso Bilbao, Andrea Calvo Pariente, Edurne Aguinaco Ortiz De Villalba. **Hospital Universitario Fundación Alcorcón. Madrid**: Pilar Sáez López, Beatriz Perdomo Ramírez, Elsa Arruti Pérez, Fátima Ngole Bebea Zamorano, Miguel Ángel Marín Aguado, Álvaro López Hualda,  María Angeles Pizarro Jaraiz, Pilar Martínez Velasco, Leandro Valdez Disla,  Victoria Sebastián Pérez, Pablo de Guinea Luengo, Isabel González Anglada, Javier Martínez Martín. **Fundación Privada Hospital Asil Granollers. Cataluña**: María Victoria Farré Mercadé, Núria Pérez Muñoz. **Hospital Universitario Príncipe de Asturias. Alcalá de Henares-Madrid:** Natalia González García, Gregorio Jiménez Díaz, María Madrigal López, María Asenjo Cambra, Fiorella Milagros Quinte Yarcuri. **Hospital Virgen de la Luz. Cuenca-Castilla-La Mancha:** María Teresa Cuerda Clares, Laura Jiménez de la Cruz, Laura Martínez Díaz. **Hospital Comarcal Monforte de Lemos. Galicia**: Javier Cambón Cotelo, Víctor Eliseo Quevedo Vila, José López Castro, Beatriz Ares Castro-Conde. **Hospital Universitario Dr. Peset. Valencia:**Carles Martínez Pérez, Pablo Correa Bellido, José Antonio Blas Dobón. **Complejo Hospitalario Torrecárdenas. Almería-Andalucía**: Juan Manuel Fernández Domínguez, Antoine Nicolas Najem Rizk, Carlos Cabeza García. **Hospital García Orcoyen. Navarra:** Javier Martínez de Morentin Garraza, Francisco Javier Artázcoz Iribarren, Amaia Arbizu Araiz, José Ramón Varela Egocheaga, Ainhoa Gordillo Santesteban, Oskia Pena Goñi, Leyre Muñoa Oteiza, Lucas Arbeloa Gutierrez, Arturo Ávila Vicioso **Hospital Universitario de Galdakao-Usansolo. Bizkaia-País Vasco**: Isidoro Calvo Lorenzo, Kattalin Iza San Juan, Xabier Uriarte Larrabeiti, Iñigo Bidea Castresana, Xabier Jiménez Urrutia, Patricia Jiménez Tellería, Andrea Vea Val, Ane Larrazabal Maruri, Sonsoles Pastor García. **Hospital Universitario Son Llàtzer. Palma de Mallorca-Baleares**: Isabel Ruiz Hernández, Juan A. Toribio Pons, Marcos Sánchez, Tomás Pujol, María Muñoz Gómez, Marina Sabater Jofre,  Bartolomé Lladó Ferrer, Violeta Sánchez María. **Hospital Nuestra Señora de Candelaria. Santa Cruz de Tenerife-Canarias:**Alicia Tejera Concepción,  Claudia Arango Salazar, Beatriz Grandal Leiros, Adela Dávila Jerez, Teresa Mendaz Siluto, Caridad Martínez Torralba,  Raquel Bachiller Caño. **Hospital Clínico Universitario Virgen de la Arrixaca**. Murcia: Elena Martínez y Juan Dionisio Avilés Hernández Moreno. **Hospital Clínico Universitario Valencia:** Amelia Pascual Ramírez y Rosana Arnau Masanet. **Hospital Son Llàtzer. Palma de Mallorca-Baleares:**Isabel María Ruiz Hernández, Juan Antonio Toribio Pons, Tomás Pujol Oliver, María Muñoz González, Marina Sabater Jofre, Denise González Acuña  y Jaime Álvarez Lastra. **Complejo Hospitalario Universitario de Cartagena. Murcia:** Carmen Allo, Inés Gil Broceño
